# False-negative malaria rapid diagnostic tests in Rwanda: impact of *Plasmodium falciparum* isolates lacking *hrp2* and declining malaria transmission

**DOI:** 10.1186/s12936-017-1768-1

**Published:** 2017-03-20

**Authors:** Christina T. Kozycki, Noella Umulisa, Stephen Rulisa, Emil I. Mwikarago, Jean Pierre Musabyimana, Jean Pierre Habimana, Corine Karema, Donald J. Krogstad

**Affiliations:** 1 Tulane University Health Science Center, New Orleans, LA USA; 2 Malaria and Other Parasitic Diseases Division, Rwanda Biomedical Centre, Ministry of Health, Kigali, Rwanda; 3 Maternal and Child Survival Programme, Jhpiego, Kigali, Rwanda; 4 School of Medicine and Pharmacy, University of Rwanda, Kigali, Rwanda; 5 National Reference Laboratory, Rwanda Biomedical Centre, Ministry of Health, Kigali, Rwanda; 6 Swiss Tropical and Public Health Institute, Basel, Switzerland; 7 University of Basel, Basel, Switzerland; 8 Quality and Equity Healthcare, Kigali, Rwanda

**Keywords:** Malaria, *Plasmodium falciparum*, Rapid diagnostic test (RDT), Histidine rich protein 2 (HRP2), *Plasmodium* lactate dehydrogenase (pLDH), Sensitivity, Polymerase chain reaction (PCR), Rwanda

## Abstract

**Background:**

Rapid diagnostic tests (RDTs) for histidine rich protein 2 (HRP2) are often used to determine whether persons with fever should be treated with anti-malarials. However, *Plasmodium falciparum* parasites with a deletion of the *hrp2* gene yield false-negative RDTs and there are concerns the sensitivity of HRP2-based RDTs may fall when the intensity of transmission decreases.

**Methods:**

This observational study enrolled 9226 patients at three health centres in Rwanda from April 2014 to April 2015. It then compared the sensitivity of RDTs based on HRP2 and the *Plasmodium* lactate dehydrogenase (pLDH) to microscopy (thick smears) for the diagnosis of malaria. PCR was used to determine whether deletions of the histidine-rich central repeat region of the *hrp2* gene (exon 2) were associated with false-negative HRP2-based RDTs.

**Results:**

In comparison to microscopy, the sensitivity and specificity of HRP2- and pLDH-based RDTs were 89.5 and 86.2% and 80.2 and 94.3%, respectively. When the results for both RDTs were combined, sensitivity rose to 91.8% and specificity was 85.7%. Additionally, when smear positivity fell from 46 to 3%, the sensitivity of the HRP2-based RDT fell from 88 to 67%. Of 370 samples with false-negative HRP2 RDT results for which PCR was performed, 140 (38%) were identified as *P. falciparum* by PCR. Of the isolates identified as *P. falciparum* by PCR, 32 (23%) were negative for the *hrp2* gene based on PCR. Of the 32 *P. falciparum* isolates negative for *hrp2* by PCR, 17 (53%) were positive based on the pLDH RDT.

**Conclusion:**

This prospective study of RDT performance coincided with a decline in the intensity of malaria transmission in Kibirizi (fall in slide positivity from 46 to 3%). This decline was associated with a decrease in HRP2 RDT sensitivity (from 88 to 67%). While *P. falciparum* isolates without the *hrp2* gene were an important cause of false-negative HRP2-based RDTs, most were identified by the pLDH-based RDT. Although WHO does not recommend the use of combined HRP2/pLDH testing in sub-Saharan Africa, these results suggest that combination HRP2/pLDH-based RDTs could reduce the impact of false-negative HRP2-based RDTs for detection of symptomatic *P. falciparum* malaria.

**Electronic supplementary material:**

The online version of this article (doi:10.1186/s12936-017-1768-1) contains supplementary material, which is available to authorized users.

## Background

Rapid diagnostic tests (RDTs) have become the focal point of malaria control. The central role of RDTs is the result of a recent paradigm shift in malaria case management, based on the World Health Organization (WHO) 2010 recommendation that all persons thought to have malaria should have their diagnosis confirmed by microscopy or an RDT before treatment [1]. However, the value of a “test before you treat” policy depends on accurate diagnosis. False-negative tests may delay the provision of life-saving treatment for individual patients and may simultaneously increase the number of persons capable of infecting mosquitoes in the community.

Although microscopy has been used most commonly to detect malaria parasites, it requires equipment, reagents and skilled microscopists [2]. Thus, in parts of sub-Saharan Africa where microscopy is inaccessible or of low quality, RDTs have become the primary tool for the parasitologic diagnosis or confirmation of malaria [3, 4]. Since 2005, the proportion of suspected malaria cases examined using a diagnostic test (microscopy or RDT) in sub-Saharan Africa rose from 36% in 2005 to 41% in 2010 and 65% in 2014. In 2014, RDTs accounted for 71% of the diagnostic tests performed in sub-Saharan Africa [3]. Given the central role RDTs now play in determining whether persons with symptoms have malaria and should be treated, it is increasingly important to understand the factors that influence their performance.

RDTs are immunochromatographic tests which detect proteins released from parasitized red blood cells. Most of the RDTs used currently to diagnose *P. falciparum* infections target HRP2 [5]. HRP2-based RDTs are specific for *P. falciparum* because *P. falciparum* is the only human parasite that produces HRP2 [3]. In contrast, RDTs targeting pan-lactate dehydrogenase (pLDH) or aldolase can detect all the *Plasmodium* species that infect humans, although they are reported to be less sensitive than HRP2-based tests, especially with low parasite densities [6, 7]. In regions where *P. falciparum* predominates and non-falciparum infections occur as mixed infections with *P. falciparum*, including most of sub-Saharan Africa, WHO generally recommends HRP2-based RDTs. In contrast, pLDH and aldolase-based RDTs are recommended in areas where non-falciparum infections predominate. Currently, WHO suggests restricting combined HRP2/pLDH RDTs to regions where *P. falciparum* and non-falciparum infections occur as single-species infections [5].

Although the sensitivity of HRP2-based RDTs has been reported as >90% for *P. falciparum*, several groups have reported decreases in the sensitivity of HRP2-based RDTs after decreases in the intensity of transmission [7–9]. For example, in Zanzibar, the sensitivity of HRP2-based RDTs in relation to thick smears fell from 93 to 79% as the percent of malaria-attributable fever episodes in the population decreased from 30 to <3% [9].

*Plasmodium falciparum* parasites without the central repeat region of the *hrp2* gene may cause false-negative RDTs [10, 11] because they fail to produce the HRP2 target molecule for HRP2-based RDTs. Isolates with *hrp2*-negative *P. falciparum* have now been identified in the blood of infected human subjects in South America, Asia and Africa [10–13]. However, despite the diagnostic threat and malaria control concerns posed by parasites without *hrp2*, there is a paucity of data on the frequency of those parasites and the factors driving (responsible for) their selection.

Preliminary studies from Mali have found seasonal fluctuations in the prevalence of false-negative RDTs and suggest the peak prevalence of *hrp2*-negative isolates is at the end of the dry season [8]. However, it is not clear whether the seasonal variation in RDT sensitivity for *P. falciparum* infection observed in Mali occurs in East Africa or elsewhere. There is also a need to determine whether *hrp2*-negative parasites can be identified using pLDH-based RDTs.

To address these knowledge gaps, this study compared the sensitivities of HRP2- and pLDH-based RDTs at sites with varying intensities of malaria transmission in Rwanda to determine whether deletions of *hrp2* were responsible for false-negative HRP2-based RDTs.

## Methods

### Study design and sites

**Fig. 1:**
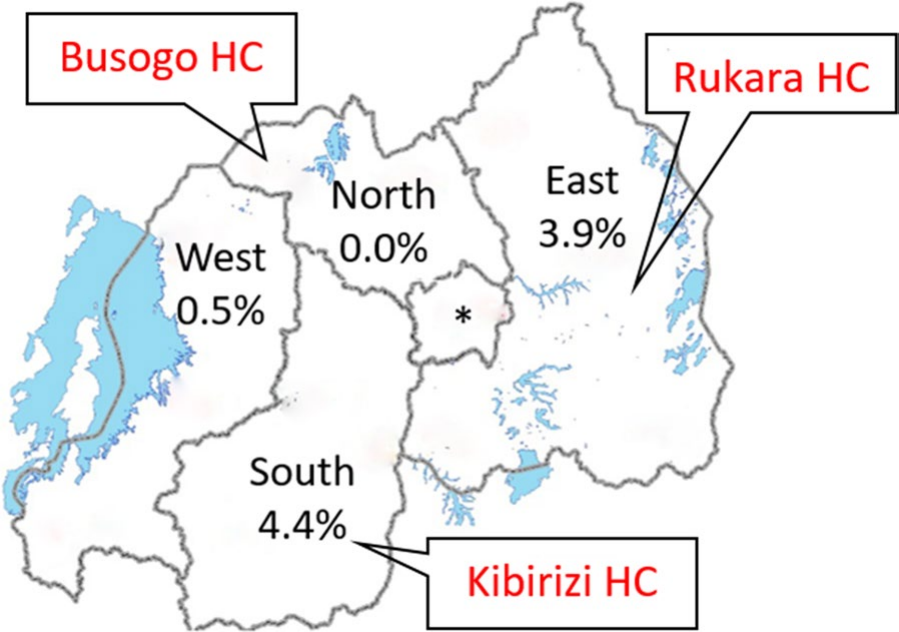
Study sites and household-level parasite prevalence in children under 5 years of age, by province. Parasite prevalence data from 2014 to 2015 DHS [16]. *Asterisk* notes the city of Kigali with a parasite prevalence of 0.0%. *HC* health center

This cross-sectional study was conducted at three primary health centres in Rwanda: Busogo Health Centre (HC) in the Musanze district of the Northern Province, Rukara HC in the Kayonza district of the Eastern Province and Kibirizi HC in the Gisagara district of the Southern Province (Fig. 1). These health centres were selected to provide sites with varying prevalences of infection consistent with different intensities of transmission. For example, in 2013, slide positivity rates for symptomatic patients were 32.0, 10.8 and 3.5% for the Kibirizi, Rukara and Busogo HCs, respectively [14]. Historically, malaria transmission has occurred year-round in Rwanda’s endemic regions with two peaks (May–June and November–December) after seasonal rains in March–April and September–October. *Plasmodium falciparum* is the predominant parasite and mosquitoes of the *Anopheles gambiae* complex are the primary vectors.

The major malaria control interventions in Rwanda are long-lasting insecticidal nets (LLINs) impregnated with pyrethroids, indoor residual spraying (IRS) with carbamates and prompt treatment of confirmed cases with artemisinin-based combination therapy (artemether–lumefantrine) [15]. Between 2009 and 2011, over 6.1 million LLINs were distributed in Rwanda (total population 10.5 million) with the goal of achieving universal coverage. Subsequently, the 2014–2015 Rwandan Demographic and Health Survey (DHS) found 83% of households owned at least one treated bed net and 43% of households had one net for every two people sleeping in the house [16]. IRS has also been implemented in districts selected by the Malaria and Other Parasitic Diseases Division (MOPDD) of the Ministry of Health including Gisagara, where the Kibirizi HC is located. During this study, two rounds of IRS (September/ October 2014 and February/March 2015) were performed in Kibirizi and coverage was reported to be greater than 98% [15]. For patients with uncomplicated malaria, artemether–lumefantrine has been the first-line treatment in Rwanda since 2006.

### Patient enrollment and study procedures

Convenience sampling was used to enroll patients who presented to these three health centres with symptoms consistent with uncomplicated malaria and were referred to the outpatient laboratory for blood smears in accordance with Rwanda malaria treatment guidelines. Study personnel were not involved in the decision to refer patients to the laboratory. Patients were excluded from this study if they were diagnosed as having severe malaria or other conditions requiring urgent diagnosis or treatment. Eligible patients were recruited Monday to Friday from 8 a.m. to 4 p.m.

At the time of enrollment, demographic and clinical information was collected and a finger-prick blood sample was obtained for thick and thin blood smears, an RDT and filter paper blots which were stored for molecular testing. Study personnel were experienced nurses and laboratory technicians trained in the performance and interpretation of blood smears and RDTs for malaria.

### Laboratory procedures

#### Rapid diagnostic tests

The RDT used in this study was the First Response^®^ Malaria pLDH/HRP2 Combo test (Premier Medical Corporation Limited, India; catalogue number I16FRC) which was provided by the Rwandan Ministry of Health. This RDT uses a buffer solution containing dye-labelled monoclonal antibodies to HRP2 and pLDH. The antigen–antibody complexes are then captured by monoclonal antibodies to the target antigens, which are immobilized on the test strip. The combo test has three lines of immobilized antibodies on the test strip: a species-specific antibody for *P. falciparum* HRP2, a genus (*Plasmodium*)-specific antibody for pLDH at a different position on the test strip and a control antibody at a third position. Testing was performed and interpreted according to the manufacturer’s instructions in the package insert. Faint bands at the test line positions were interpreted as positive [9, 17].

#### Microscopy

Thick and thin blood smears were collected on the same slide, air-dried and stained with 10% Giemsa for 10 min after the thin smears had been fixed with methanol. Thick smears were examined under oil immersion (1000× magnification) by two independent laboratory technicians. Asexual parasitaemia at any parasite density was reported as a positive smear. Blood smears were considered negative if no parasites were observed after the examination of 100 oil immersion fields. Disparities in slide readings (positive vs negative) and differences >30% in parasite densities were resolved by a third reader. Note that study microscopists did not have access to the subjects’ RDT results or previous microscopy results. All laboratory technicians were qualified at the diploma level or above in the medical laboratory sciences.

#### Filter paper blots

Blood samples collected on filter paper (Whatman 3 MM) were air-dried thoroughly, placed in individual sealed plastic bags with desiccant and stored at room temperature prior to molecular analysis by PCR.

#### Amplification of the central repeat region of the *hrp2* gene (exon 2) and 18S rRNA parasite DNA sequences

PCR was performed to test for *Plasmodium* DNA in samples that were negative using the HRP2 RDT but positive by microcopy or the pLDH RDT. Within 3 months of sample collection, DNA was isolated from dried blood spots on filter papers using six 3 mm punches and the QIAamp DNA mini kit (Qiagen, Germantown, MD). Positive and negative controls were used with each round of PCR. After PCR, amplicons were visualized by electrophoresis using 2% agarose gels stained with ethidium bromide.

#### PCR protocol

**Fig. 2:**
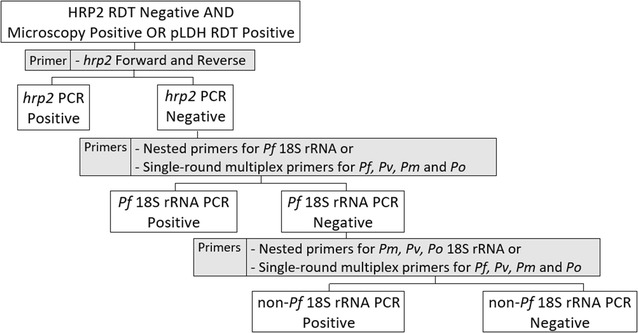
PCR amplification of samples negative with the HRP2 RDT which were positive by microscopy or by pLDH RDT. PCR was performed to test for *Plasmodium* DNA in samples that were negative by HRP2 RDT but positive by either microcopy or by pLDH RDT. PCR was performed using a tiered, 3-stage approach. *Rows 2–4* of the flowchart reflect PCR performed in stages 1–3, respectively. The primers used at each stage are listed in the *gray boxes* to the *right* of the flowchart. If a sample was identified as positive by PCR at any stage, no further PCR testing was pursued. Samples positive by PCR for the *hrp2* gene were considered *P. falciparum* isolates containing the *hrp2* gene. Samples negative by PCR for the *hrp2* gene and positive by PCR for *Pf* 18S rRNA were considered *P. falciparum* isolates lacking the *hrp2* gene. Samples negative by PCR for the *hrp2* gene and *Pf* 18S rRNA but positive by PCR for *Pv, Pm* or *Po* 18S rRNA were considered non-*Pf* malaria. Samples negative by PCR for the *hrp2* gene, *Pf* 18S rRNA and *Pv, Pm* and *Po* 18S rRNA were considered negative for Plasmodium DNA. *Pf Plasmodium falciparum, Pv P. vivax, Pm P. malariae, Po P. ovale*

A sequential, 3-step approach to PCR was used to assign each sample to one of four categories: (1) DNA from *P. falciparum* with the *hrp2* gene, (2) DNA from *P. falciparum* without the central histidine-rich repeat region of the *hrp2* gene, (3) DNA from *Plasmodium* other than *P. falciparum* and (4) samples without *Plasmodium* DNA. This stepwise protocol is depicted in Fig. 2 and described below.

For samples negative with the HRP2 RDT but positive by microcopy or the pLDH RDT, primers for the conserved 5′ and 3′ regions of the *hrp2* gene (Additional file 1: Table S1) were used to amplify the histidine-rich central repeat region of the *hrp2* gene [10]. Samples positive for the *hrp2* gene with this PCR were designated as *P. falciparum* with the *hrp2* gene and no further PCR testing was performed.

Samples PCR-negative for the *hrp2* gene were examined by PCR using primers for conserved regions of *P. falciparum* 18S rRNA [18, 19]. Samples positive in this PCR for *Pf* 18S rRNA were designated as *P. falciparum* without the *hrp2* gene and no further testing was performed.

In contrast, samples negative for *P. falciparum* 18S rRNA were re-examined by PCR using primers for conserved regions of 18S rRNA in *Plasmodium vivax, Plasmodium malariae* and *Plasmodium ovale* [18, 19]. Samples positive using primers for 18S rRNA from *P. vivax*, *P. malariae* or *P. ovale* were designated non-*P. falciparum* malaria parasites and no further testing was performed. Samples negative in this PCR were considered negative for *Plasmodium* DNA and no further testing was performed.

The primers used are listed in Additional file 1 and a sample gel is presented in Additional file 2. Because the prevalence of non-falciparum *Plasmodium* parasites was greater than anticipated, amplification of 18S rRNA was changed from nested PCR described by Singh et al. [19] to multiplex PCR capable of detecting *P. falciparum, P. vivax, P. malariae* and *P. ovale* in a single-round of amplification to minimize the number of rounds of amplification required for species identification [18]. Primers targeting 18S rRNA have been reported to detect parasitemia at parasite densities as low as 1 parasite per microlitre [18, 20].

### Statistical methods

Microsoft Excel was used for data entry and Stata (version 13) for analysis. The sensitivity, specificity and positive and negative predictive values of the HRP2 and pLDH RDTs were compared to microscopy (thick smears) which was used as the reference standard. RDT results were considered true positives or true negatives if they were concordant with microscopy. Negative RDT results were considered false-negatives if microscopy was positive. Positive RDTs were considered false-positives if microscopy was negative. Differences with a probability of less than 0.05 (*P* < 0.05) were accepted as significant.

## Results

**Fig. 3:**
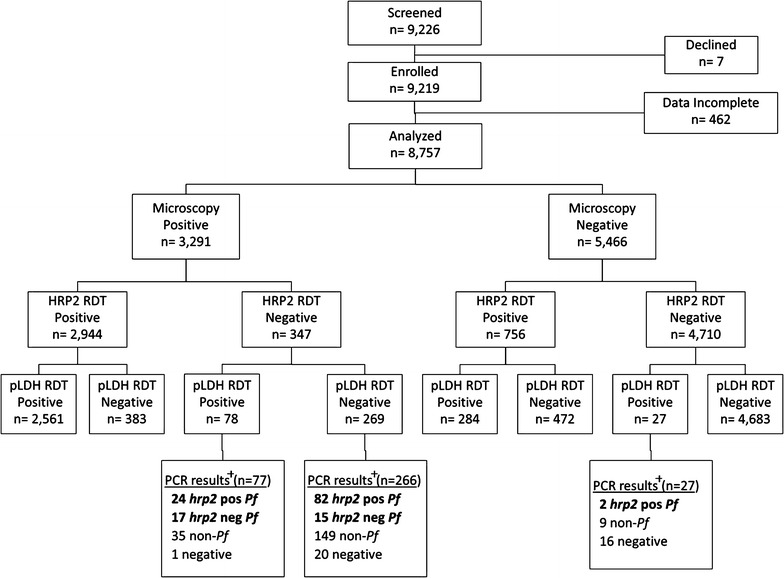
Laboratory results for patients enrolled at all three health centers. Samples positive by PCR targeting the *hrp2* gene were labelled *hrp2* pos *Pf*, samples negative by PCR targeting the *hrp2* gene and positive by PCR targeting *Pf* 18S rRNA were labelled *hrp2* neg *Pf*, samples negative by PCR targeting the *hrp2* gene and *Pf* 18S rRNA but positive by PCR targeting *Pv, Pm* or *Po* 18S rRNA were labelled non-*Pf* and samples negative by PCR targeting the *hrp2* gene, *Pf* 18S rRNA and *Pv, Pm* and *Po* 18S rRNA were labelled negative. *Pf Plasmodium falciparum, Pv P. vivax, Pm P. malariae, Po P. ovale*, *RDT* rapid diagnostic test, *HRP2* histidine rich protein 2, *pLDH Plasmodium* lactate dehydrogenase, *PCR* polymerase chain reaction, *Pf Plasmodium falciparum*, *non-Pf* non-*Plasmodium falciparum*

The flow of patients through the study is outlined in Fig. 3 and Table 1 provides a summary of baseline characteristics for the patients enrolled at each site. Of the 9226 patients screened, seven did not provide informed consent for their participation. Of the 9219 patients who consented to participate in the study, 462 were not included in the analysis because of incomplete test results (invalid or unrecorded RDT or microscopy results, other missing data or unreadable blood smears). Thus, 8757 sets of paired RDT and microscopy results for 8757 patients were included in the analysis (Fig. 3).

**Table 1:** Baseline characteristics

Characteristic	Sites		
	Kibirizi n = 5148	Rukara n = 2696	Busogo n = 913
Altitude (m)	1718	1591	2247
Average annual rainfall (mm) [36]	81	75	129
EIR^a^ [15]	–	20.9	<1^b^
IRS with carbamates	Sep ‘14; Feb ‘15		
Dates of study	Apr ‘14–Apr ‘15	Oct ‘14–Mar ‘15	Nov ‘14–Apr ‘15
Mean age (95% CI)	22.3 (21.7–22.8)	20.8 (20.1–21.5)	20.5 (19.5–21.6)
Age <5 years, % (95% CI)	19.4 (18.4–20.6)	17.5 (16.2–19.0)	22.3 (19.8–25.2)
SPR if age <5 years, % (95% CI)	32.1 (29.1–35.1)	41.6 (37.2–46.1)	2.4 (0.3–4.6)
SPR if age ≥5 years, % (95% CI)	36.0 (34.5–37.5)	55.3 (53.2–57.4)	11.6 (9.2–13.9)

*EIR* entomological inoculation rate, *IRS* indoor residual spraying, *CI* confidence interval, *SPR* slide positivity rate

^a^EIR defined as the number of infective *Anopheles* mosquito bites per person per year

^b^Data provided from closest sentinel site, Bungwe (no sentinel site in Busogo HC’s district)

### Study sites

**Fig. 4:**
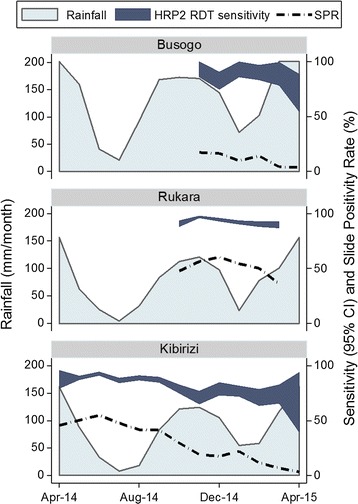
HRP2 RDT sensitivity, slide positivity rate and rainfall by study site and month. Rainfall is plotted in millimeters per month on the *left y axis*. Sensitivity and slide positivity rate are plotted on the *right y axis*. The navy band represents the 95% confidence interval for sensitivity. The *dashed line* represents the slide positivity rate. There was significant variation in HRP2 RDT sensitivity across the study sites and a decline in RDT sensitivity was noted following a decline in malaria transmission. In Rukara, monthly estimates of HRP2 RDT sensitivity ranged from 90 to 97%. Conversely, in Kibirizi, a site subject to two rounds of IRS during the study period, the sensitivity of the HRP2 RDT declined from 88 to 67% as the slide positivity rate fell from 46 to 3%. SPR slide positivity rate; *CI* confidence interval

Patients were enrolled from three sites across Rwanda which vary in malaria endemicity. When the study sites were selected, malaria transmission was believed to be lowest in Busogo and highest in Kibirizi. The rationale for enrolling subjects in Kibirizi for 12 months was to determine whether RDT sensitivity varied seasonally. However, Kibirizi HC is within a district targeted for IRS by Rwanda’s MOPDD and two rounds of IRS were performed in Kibirizi during this study: in September 2014 and February 2015. Overall, the fractions of referred patients with positive thick smears for *Plasmodium* species were 10% (87/913) at Busogo, 35% (1778/5148) at Kibirizi and 53% (1426/2696) at Rukara. At the Kibirizi HC, the monthly slide positivity rate declined from 46% in April 2014 to 3% in April 2015 after two rounds of IRS (Fig. 4).

### Sensitivity and specificity: HRP2 RDT vs combined HRP2 + pLDH RDT

Among the 8757 patients for whom microscopy and RDT test results were available, 3291 (38%) were positive for *Plasmodium* species by thick smear. Among those specimens, the HRP2 RDT identified 2944 positives and the pLDH-based RDT identified 2639 positives. The overall sensitivities and specificities of the RDTs in comparison to microscopy were 89.5% (95% confidence interval [CI] 88.4–90.5) and 86.2% (95% CI 85.2–87.1) for the HRP2 RDT and 80.2% (95% CI 78.8–81.5) and 94.3% (95% CI 93.7–94.9) for the pLDH RDT, respectively. The pLDH RDT detected 78 microscopy-positive samples that were missed by the HRP2 RDT. Thus, when the pLDH RDT results were considered with the HRP2 RDT results, RDT sensitivity increased from 89.5 to 91.8% (95% CI 90.8–92.7) and there was no significant difference (P = 0.29) in RDT specificity. When the HRP2 and pLDH RDTs were considered together, RDT specificity was 85.7% (95% CI 84.7–86.6) (Table 2).

**Table 2:** RDT sensitivity and specificity for the diagnosis of malaria compared to microscopy as the gold standard

RDT target	Sensitivity, % (95% CI)			All sites
	Kibirizi	Rukara	Busogo	
HRP2 RDT	86.2 (84.5–87.8)	93.6 (92.2–94.8)	87.4 (78.5–93.5)	89.5 (88.9–90.0)
pLDH RDT	81.4 (79.5–83.2)	79.9 (77.8–82)	59.8 (48.7–70.1)	80.2 (79.5–80.9)
HRP2 + pLDH RDT	90.5 (89.0–91.8)	93.8 (92.4–95.0)	87.4 (78.5–93.5)	91.8 (91.3–92.3)

RDT target	Specificity, % (95% CI)			All sites
	Kibirizi	Rukara	Busogo	
HRP2 RDT	84.7 (83.5–85.9)	83.4 (81.2–85.4)	96.2 (94.7–97.4)	86.2 (85.7–86.6)
pLDH RDT	92.7 (91.8–93.6)	95.4 (94.1–96.5)	99.0 (98.1–99.6)	94.3 (94.0–94.6)
HRP2 + pLDH RDT	84.0 (82.7–85.2)	83.3 (81.1–85.3)	96.2 (94.7–97.4)	85.7 (85.2–86.1)

*CI* confidence interval, *RDT* rapid diagnostic test, *HRP2* histidine rich protein 2, *pLDH Plasmodium* lactate dehydrogenase

### Variation in sensitivity of the HRP2 RDT and the slide positivity rate

Variations in the sensitivity of the HRP2 RDT were noted by site and month (Fig. 4). The sensitivity of the HRP2 RDT was greatest at Rukara (90–97%), the site with the highest fraction of positive blood smears (slide positivity rate). In contrast, at Kibirizi, when the smear positivity rate decreased from 55% in June 2014 to 3% in April 2015, the sensitivity of the HRP2 RDT fell from 93 to 67% (Chi square for trend = 37.2, P < 0.001). At Busogo, the monthly sensitivity of the HRP2 RDT ranged from 71 to 93% (Chi square for trend = 0.4, P = 0.522).

### False-negative HRP2 RDTs and PCR for the *hrp2* gene

PCR studies were performed for 343 of the 347 samples with false-negative HRP2 RDTs (Fig. 3). Of the 343 samples examined, *Plasmodium* DNA was detected using primers for the histidine-rich central repeat region of the *hrp2* gene or conserved loci in *Plasmodium* 18S rRNA [10, 18, 19] in 322 (94%) of samples. Of these 322 samples, 138 (43%) were positive by PCR for *P. falciparum* DNA using primers for the histidine-rich central repeat region of the *hrp2* gene or *P. falciparum* 18S rRNA and 184 (57%) were positive only by PCR for 18S rRNA from non-falciparum species. Of the 138 *P. falciparum* samples with false-negative RDTs, 106 (77%) were positive for the *P. falciparum hrp2* gene and 32 (23%) were negative on PCR for the central repeat region of the *hrp2* gene but positive for *P. falciparum* 18S rRNA (consistent with *hrp2* deletion).

**Fig. 5:**
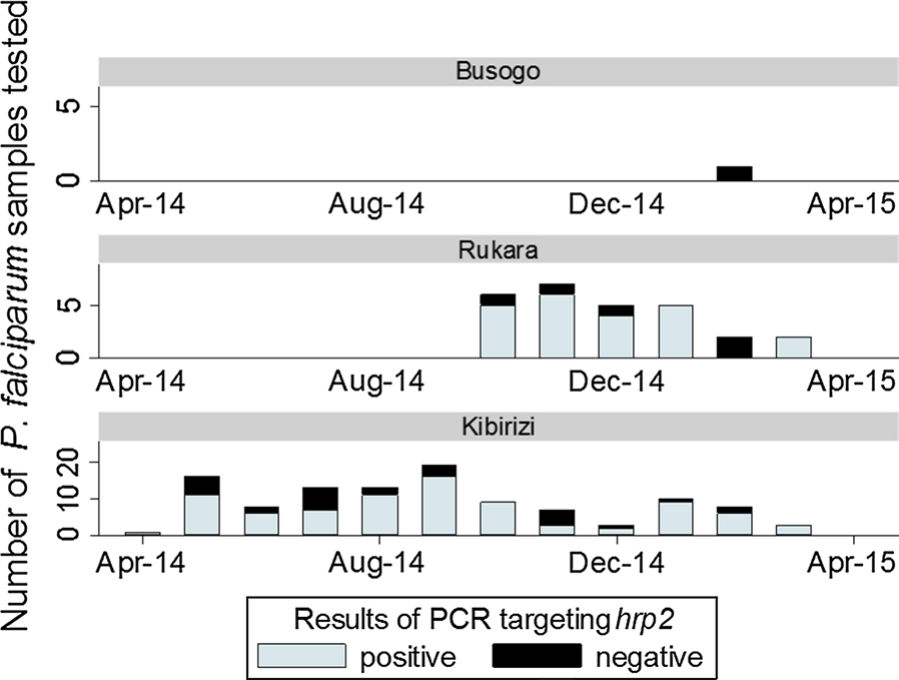
*hrp2* PCR for *P. falciparum* isolates with false-negative HRP2 RDTs. Of the 138 *P. falciparum* samples with false-negative HRP2 RDTs, 106 were positive by PCR for the *P. falciparum hrp2* gene (*light blue bars*) and 32 were negative by PCR for the central repeat region of the *hrp2* gene (*black bars*). In Kibirizi, improved malaria control was not associated with an increased frequency of false-negative RDTs due to *hrp2*-negative *P. falciparum* isolates

In Kibirizi, PCR for the *hrp2* gene was negative for 26 of the 110 (24%) of the *P. falciparum* infections with false-negative RDTs and the proportion of *hrp2*-negative isolates did not increase as the slide positivity rate decreased (Fig. 5).

### pLDH RDT detection of samples with false-negative HRP2 RDTs

The pLDH RDT was positive for 41 of the 138 (30%) *P. falciparum* infections with false-negative HRP2 RDTs. Notably, the pLDH RDT was positive for the majority (53%) of the *P. falciparum* samples with false-negative HRP2 RDTs that were negative by PCR for the *hrp2* gene.

### pLDH RDT results for samples positive by microscopy

**Fig. 6:**
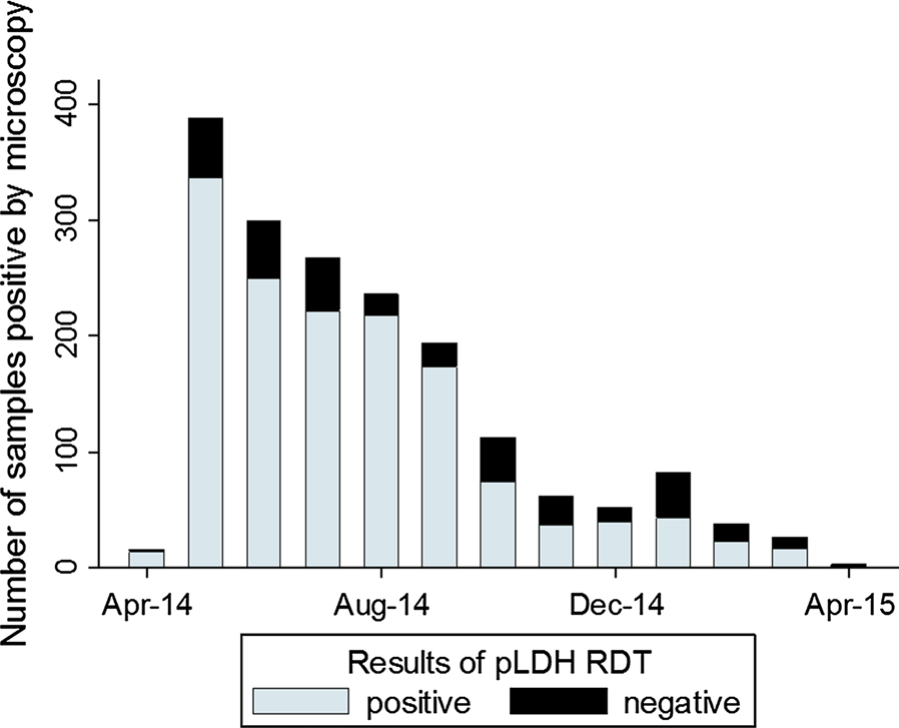
pLDH RDT results for microcopy positive samples in Kibirizi. Of the 1778 microscopy positive samples from Kibirizi, 1447 samples were positive by the pLDH RDT (*light blue bars*) and 331 samples were negative by the pLDH RDT (*black bars*). Improved malaria control was associated with an increased in the proportion of the microscopy positive samples that were negative by the pLDH RDT

In Kibirizi, the proportion of microscopy positive samples that were negative by pLDH RDT increased as slide positivity decreased (Fig. 6). The proportion of microscopy positive and pLDH negative samples rose from 13.9% (95% CI 12.7–16.0) during April to August 2014 to 38.6% (95% CI 32.1–45.6) during December 2014 to April 2015.

### pLDH RDT positive samples negative by microscopy and HRP2 RDT

PCR studies were also performed on 27 samples positive the pLDH RDT but negative by microscopy and the HRP2 RDT. Of these 27 samples, 2 were positive by PCR for *hrp2* and 9 were positive only by PCR for 18S rRNA from non-falciparum species.

## Discussion

In sub-Saharan Africa, HRP2 RDTs are the test used most commonly for parasitologic confirmation of malaria before treatment [5]. However, several reports have noted significant declines in the sensitivity of HRP2 RDTs after declines in the intensity of transmission [7–9]. In Mali, preliminary studies found seasonal declines in RDT sensitivity were associated with peaks in the prevalence of *hrp2*-negative *P. falciparum* isolates at the end of the dry season [8]. It is not clear if the association between declining RDT sensitivity and increasing prevalence of *hrp2*-negative isolates observed in Mali occurs elsewhere.

Thus, this study compared the sensitivity of HRP2 RDTs at 3 sites with varying intensities of transmission in Rwanda to determine whether deletions of *hrp2* were responsible for false-negative HRP2-based RDTs. RDT performance was examined in relation to microscopy which was considered the gold standard. Samples with false-negative HRP2 RDTs (positive smear, negative RDT) were re-examined using PCR to test for the *hrp2* gene.

Consistent with previous reports, this study found that HRP2-based RDTs were more sensitive than pLDH-based RDTs, although less specific [6, 7, 17]. However, when the HRP2 and pLDH RDTs were considered together, sensitivity increased slightly, without a decline in specificity. Using both RDTs, sensitivity was 91.8% and specificity was 85.7%.

Notably, the sensitivity of HRP2-based RDTs varied across the study sites and there was a decrease in the sensitivity of the HRP2 RDT after a fall in malaria transmission. In Rukara, monthly estimates of HRP2 RDT sensitivity ranged from 90 to 97%. Conversely, in Kibirizi, the sensitivity of the HRP2 RDT declined from 88 to 67% after two rounds of IRS as slide positivity rate for symptomatic patients fell from 46 to 3%.

Although IRS appeared to reduce the incidence of malaria in Kibirizi, a recent study in an area of The Gambia with high LLIN coverage found no additional benefit from adding IRS. Of note, in The Gambia, ≥93% LLIN coverage was achieved for all sleeping spaces and IRS coverage was 83–86%. [21]. In contrast, the 2014–2015 Rwandan DHS found that IRS achieved >98% coverage of targeted areas while only 81% of households had at least one LLIN and 43% of households had an LLIN for every two persons [16].

Potential explanations for false-negative HRP2 RDTs and the decline of RDT sensitivity in Kibirizi include: loss (deletion) of the *hrp2* gene and low parasite densities [6, 10, 11].

Parasites lacking the *hrp2* gene are a potential source of false-negative HRP2 RDTs. The *hrp2* gene is absent in *P. falciparum* isolates with *hrp2* gene deletions and in non-falciparum *Plasmodium* parasites. PCR analysis was used to identify isolates without the *hrp2* gene and to confirm the presence *P. falciparum* DNA. Of 138 *P. falciparum* infections with false-negative HRP2 RDTs, 32 were negative by PCR for *hrp2* (consistent with deletion of the *hrp2* gene).

*Plasmodium falciparum* isolates lacking the *hrp2* gene appear to be a significant source of false-negative RDTs in Rwanda. However, in this study, most *P. falciparum* isolates lacking the *hrp2* gene were detected by the pLDH-based RDT and improved malaria control was not associated with an increased frequency of false-negative RDTs due to *hrp2*-negative *P. falciparum* isolates (Fig. 5).

For the majority (106/138 = 77%) of *P. falciparum* samples with false-negative HRP2 RDT results, PCR for *hrp2* was positive. *P. falciparum* isolates containing the *hrp2* gene may produce a false-negative RDT if the parasite density is below the threshold for RDT detection. Although this study lacks quantitative data on parasite density, a positive pLDH RDT may provide a crude estimate of the parasite density (≥200–1000) parasites per microlitre [22]. Because HRP2-based RDTs are more sensitive than pLDH-based RDTs at low parasite densities, a positive pLDH RDT suggests the parasite density was at or above the threshold for HRP2 RDT detection [6, 7, 23, 24]. Conversely, microscopy positive/pLDH negative *P. falciparum* samples may reflect low density infections.

In this study, *P. falciparum* infections with parasite densities below the threshold for detection may be responsible for many of the false-negative RDTs. Of the 106 *P. falciparum* isolates with false-negative HRP2 RDTs and the *hrp2* gene (confirmed by PCR), most (77%) were negative by pLDH RDT. Additionally, in Kibirizi, the proportion of microscopy positive/pLDH RDT negative samples increased as the slide positivity rate fell (Fig. 6). The proportion of microscopy positive/pLDH negative samples rose from 13.9% (95% CI 12.7–16.0) during April to August 2014 to 38.6% (95% CI 32.1–45.6) during December 2014 to April 2015. This increase in the proportion of microscopy positive/pLDH negative samples may reflect an increase in the proportion of low density infections. Thus, the pLDH RDT data suggest that a decline in parasite density may have contributed to the decrease in HRP2 RDT sensitivity as malaria control improved in Kibirizi.

Other potential causes for false-negative RDTs which were not examined in this study include partial deletions of the *hrp2* gene, prozone effects due to excess antigen, sequence variability of *P. falciparum hrp2* and circulating antibodies to HRP2 which have been reported to interfere with RDT detection of HRP2 [11, 25–28]. While the primers used in this study amplified only exon 2 of the *hrp2* gene, the *hrp2* gene is also known to have chromosomal breaking points outside exon 2 [28].

There have been several reports of prozone-like effects with HRP2-based RDTs in patients with hyperparasitaemia. Although the mechanism of prozone-like effects for antigen detection tests is not well defined, one plausible explanation is that the amount of antigen may exceed the binding capacity of the dye-labelled antibodies used for antigen detection. In this situation, unlabelled target antigen reaches the test strip and saturates the binding capacity of the capture antibodies affixed to the test strip. As a result, antigen captured by dye-labelled antibodies may be unable to bind to the test strip to form a visible band [29]. However, false-negative HRP2 test lines attributed to the prozone effect have been described only in samples with ≥288,000 parasites/µL [30, 31]. While this study lacks data on parasite density, results of previous studies suggest that hyperparasitaemia is unlikely to have been a significant cause of false-negative RDTs in this study [31].

Of the 343 samples with false-negative HRP2 RDTs that were tested by PCR, 21 were negative by PCR for both *hrp2* and 18S rRNA of the four *Plasmodium* parasites known to cause human infection. Sub-optimal PCR sensitivity may have occurred as a result of inadequate DNA sample, degradation of DNA sample, presence of PCR inhibitors and deletion or mutation of the targeted DNA [20]. Other possible explanations for these discrepancies include false-positive microscopy results and pLDH RDT cross-reactivity with other infectious agents, such as African trypanosomes [32].

Importantly, there is potential for confusion about the RDT used in this study because Premier Medical Corporation Ltd. submitted two different products with the name “First Response^®^ Malaria Ag. pLDH/HRP2 Combo Card Test” to WHO for testing. The RDT used in this study, catalogue number I16FRC, was tested in rounds 1, 2 and 5. However, a different product was tested in round 6 (catalogue number PI16FRC). Product I16FRC did not meet WHO recommended procurement criteria during round 5 of WHO RDT lot testing because the panel detection score (PDS) for *P. vivax* at 200 parasites per microlitre was 74.5 (below the WHO criterion of ≥75). In contrast, product I16FRC had a satisfactory PDS score of 85.0 for *P. falciparum* (please note that PDS is not equivalent to sensitivity) [6]. These data are available at: http://www.rdt-interactive-guide.org/ [33]. Because *P. falciparum* is the predominant species in Rwanda, the marginally low sensitivity of this RDT for *P. vivax* would not be expected to have a significant impact of the finding of this study [5].

Finally, the authors recognize the lack of testing for additional single-copy genes is a theoretical limitation because the reported sensitivity of PCR is greater for multi-copy genes (18S rRNA) than single copy genes (*hrp2*) (1 vs 10–100 parasite per microlitre) [34]. However, several factors suggest the parasite densities of the *P. falciparum* samples that were negative by PCR for *hrp2* were above the threshold for detection by PCR for single-copy genes: all patients had symptoms consistent with malaria infection, DNA was extracted within 3 months of sample collection and most *P. falciparum* isolates without the *hrp2* gene were detected by the pLDH RDT. Most symptomatic individuals in malaria-endemic areas have parasite densities ≥1000 parasites per microlitre [35] and prompt extraction of DNA limits the time for DNA degradation which may disproportionately reduce the sensitivity of PCR for single-copy genes. Additionally, based on the results from round 5 of WHO RDT quality testing, a positive pLDH RDT suggests the parasite density was above the threshold for detection by PCR for single-copy genes (≥200 parasites per microlitre). The pLDH RDT of the First Response^®^ Malaria pLDH/HRP2 Combo test (catalogue number I16FRC) had a sensitivity of 31% for wild-type (clinical) *P. falciparum* smear-positive samples with 200 parasites per microlitre. In contrast, with parasite densities of 2000 per microlitre, the sensitivity of the pLDH RDT was 100% [22].

Ultimately, the factors driving the decline in RDT sensitivity as malaria control improves are not clear. If parasite density declines as malaria control improves, the decrease in RDT sensitivity could be driven in part by an increase in the number of infections with parasite densities below the RDT threshold for detection. In addition, there are concerns that *hrp2*-negative parasites may have an increased impact on RDT performance as malaria control improves [10, 11]. Conversely, in high transmission settings, *hrp2* negative parasites may have less impact on RDT sensitivity because individuals are commonly infected with more than one *P. falciparum* parasite strain (genotype) and the RDT will yield a true-positive result if any one parasite (genotype) is *hrp2*-positive [10]. However, the number of parasite genotypes infecting individual subjects, the multiplicity of infection (MOI), declines as malaria control improves. Conversely, individuals infected by only a single parasite genotype negative for the *hrp2* gene will produce false-negative results when tested with an HRP2-based RDT [10]. In the present study, the decrease in HRP2 RDT sensitivity as malaria control improved was not associated with an increased frequency of *hrp2*-negative *P. falciparum* isolates. However, it may have been associated with an increase in the frequency of low density infections.

## Conclusions

This study provides new information on the performance of RDTs in Rwanda and supports previously raised concerns that the sensitivity of HRP2 RDTs may decline as malaria control improves. In addition, the results of this study suggest that the use of three-band RDTs (HRP2, pLDH and control bands) may improve sensitivity without decreasing specificity. This study also found that *P. falciparum* isolates lacking the *hrp2* gene are an important source of false-negative HRP2 RDTs in Rwanda. Further investigations are warranted to better define the prevalence of these isolates and the factors responsible for the decline in RDT sensitivity as malaria control improves.

## Additional files

**Additional file 1.** Primer sequences used to amplify *hrp2* and *P. falciparum*, *P. vivax*, *P. malariae* and *P. ovale* 18S rRNA, expected product sizes and PCR conditions.

**Additional file 2.** PCR products visualized on an agarose gel. DNA is from two thick-smear positive subjects with negative HRP2-based RDTs. Lane 1: 100 bp marker. Lane 2: PCR targeting hrp2 with DNA from subject #1 (shows amplicon of expected size of ~900 bp). Lanes 3–5: PCR with DNA from subject #2. Lane 3 shows the absence of hrp2 amplicons. Lane 4 shows results of multiplex PCR for 18S rRNA with an amplicon of 276 bp (expected size for *P. falciparum*). Lane 5 shows results of nested PCR with species-specific primers for *P. falciparum* 18S rRNA with an amplicon of the expected size of 205 bp.

## Abbreviations

RDT: rapid diagnostic test
WHO: World Health Organization
HRP2: histidine-rich protein 2
pLDH: *Plasmodium* lactate dehydrogenase
HC: health centre
LLINs: long-lasting insecticidal nets
IRS: indoor residual spraying
DHS: Demographic and Health Survey
MOPDD: Malaria and Other Parasitic Diseases Division
PCR: polymerase chain reaction
CI: confidence interval
