# Supplementary material for: False-negative malaria rapid diagnostic tests in Rwanda: impact of Plasmodium falciparum isolates lacking hrp2 and declining malaria transmission
Source: Malar J. 2017 Mar 20;16:123. doi: 10.1186/s12936-017-1768-1 (PMC5359811; doi:10.1186/s12936-017-1768-1)
Supplement: Supplementary file 1 — Additional file 1. Primer sequences used to amplify hrp2 and P. falciparum, P. vivax, P. malariae and P. ovale 18S rRNA, expected product sizes and PCR conditions. [file 12936_2017_1768_MOESM1_ESM.docx]

| **Primers** | **Sequence** | **Size** | **PCR conditions** |
| --- | --- | --- | --- |
| **HRP2 [10]** |  |  |  |
| HRP2 F | 5' ATT CCG CAT TTA ATA ATA ACT TGT GTA GC 3' | 74 – 968 bp | 94°C for 4 min; 40 cycles of 94°C for 30 sec, 50°C for 60 sec, and 72°C for 60 sec; 72°C for 7 min |
| HRP2 R | 5′ ATG GCG TAG GCA ATG TGT GG 3′ |  |  |
| **Single round multiplex PCR [18]** | | |  |
| Conserved *Plasmodium* R | 5' GTA TCT GAT CGT CTT CAC TCC C 3' |  | 94°C for 4 min; 45 cycles of 94°C for 45 sec, 60°C for 90 sec, and 72°C for 60 sec; 72°C for 7 min |
| Pf F | 5' AAC AGA CGG GTA GTC ATG ATT GAG 3′ | 276 bp |  |
| Pv F | 5' CGG CTT GGA AGT CCT TGT 3' | 300 bp |  |
| Po F | 5' CTG TTC TTT GCA TTC CTT ATG C 3′ | 375 bp |  |
| Pm F | 5' CGT TAA GAA TAA ACG CCA AGC G 3′ | 412 bp |  |
| **Nested PCR [19]** | | |  |
| Nest 1 - Genus-specific | | |  |
| rPLU1 | 5′ TCA AAG ATT AAG CCA TGC AAG TGA 3′ |  | 94°C for 4 min; 35 cycles of 94°C for 30 sec, 55°C for 60 sec, and 72°C for 60 sec; 72°C for 7 min |
| rPLU5 | 5′ CCT GTT GTT GCC TTA AAC TCC 3′ |  |  |
| Nest 2 - Genus-specific | | |  |
| rPLU 3 | 5′ TTT TTA TAA GGA TAA CTA CGG AAA AGC TGT 3′ | 240 bp | 94°C for 4 min; 35 cycles of 94°C for 30 sec, 62°C for 60 sec, and 72°C for 60 sec; 72°C for 7 min |
| rPLU 4 | 5′ TAC CCG TCA TAG CCA TGT TAG GCC AAT ACC 3′ |  |  |
| Nest 2 - Species-specific | | |  |
| rFAL 1 | 5′ TTA AAC TGG TTT GGG AAA ACC AAA TAT ATT 3′ | 205 bp | 94°C for 4 min; 35 cycles of 94°C for 30 sec, 58°C for 60 sec, and 72°C for 60 sec; 72°C for 7 min |
| rFAL2 | 5′ ACA CAA TGA ACT CAA TCA TGA CTA CCC GTC 3′ |  |  |
| rVIV1 | 5′ CGC TTC TAG CTT AAT CCA CAT AAC TGA TAC 3′ | 117 bp |  |
| rVIV2 | 5′ ACT TCC AAG CCG AAG CAA AGA AAG TCC TTA 3′ |  |  |
| rMAL1 | 5′ ATA ACA TAG TTG TAC GTT AAG AAT AAC CGC 3′ | 144 bp |  |
| rMAL2 | 5′ AAA ATT CCC ATG CAT AAA AAA TTA TAC AAA 3′ |  |  |
| rOVA1 | 5′ ATC TCT TTT GCT ATT TTT TAG TAT TGG AGA 3′ | 787 bp |  |
| rOVA2 | 5′ GGA AAA GGA CAC ATT AAT TGT ATC CTA GTG 3′ |  |  |
