# Supplementary figures and images for: False-negative malaria rapid diagnostic tests in Rwanda: impact of Plasmodium falciparum isolates lacking hrp2 and declining malaria transmission
Source: Malar J. 2017 Mar 20;16:123. doi: 10.1186/s12936-017-1768-1 (PMC5359811; doi:10.1186/s12936-017-1768-1)

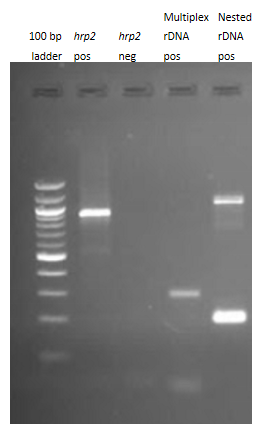

Supplement: Supplementary file 2 — Additional file 2. PCR products visualized on an agarose gel. DNA is from two thick-smear positive subjects with negative HRP2-based RDTs. Lane 1: 100 bp marker. Lane 2: PCR targeting hrp2 with DNA from subject #1 (shows amplicon of expected size of ~900 bp). Lanes 3–5: PCR with DNA from subject #2. Lane 3 shows the absence of hrp2 amplicons. Lane 4 shows results of multiplex PCR for 18S rRNA with an amplicon of 276 bp (expected size for P. falciparum). Lane 5 shows results of nested PCR with species-specific primers for P. falciparum 18S rRNA with an amplicon of the expected size of 205 bp. [file 12936_2017_1768_MOESM2_ESM.docx]
